# Combination of artificial intelligence‐based endoscopy and miR148a methylation for gastric indefinite dysplasia diagnosis

**DOI:** 10.1002/jcla.24122

**Published:** 2021-11-22

**Authors:** Yoshiyuki Watanabe, Ritsuko Oikawa, Shuhei Agawa, Yasumasa Matsuo, Ichiro Oda, Seiji Futagami, Hiroyuki Yamamoto, Tomohiro Tada, Fumio Itoh

**Affiliations:** ^1^ Division of Gastroenterology and Hepatology Department of Internal Medicine St. Marianna University School of Medicine Kanagawa Japan; ^2^ Department of Internal Medicine Kawasaki Rinko General Hospital Kanagawa Japan; ^3^ Division of Gastroenterology Department of Internal Medicine Nippon Medical School Tokyo Japan; ^4^ Department of Bioinformatics St. Marianna University Graduate School of Medicine Kanagawa Japan; ^5^ Tada Tomohiro Institute of Gastroenterology and Proctology Saitama Japan

**Keywords:** artificial intelligence, endoscopy, DNA methylation, gastric indefinite dysplasia, gastric cancer, endoscopy, molecular markers

## Abstract

**Background and Aim:**

Gastrointestinal endoscopy and biopsy‐based pathological findings are needed to diagnose early gastric cancer. However, the information of biopsy specimen is limited because of the topical procedure; therefore, pathology doctors sometimes diagnose as gastric indefinite for dysplasia (GIN).

**Methods:**

We compared the accuracy of physician‐performed endoscopy (trainee, *n* = 3; specialists, *n* = 3), artificial intelligence (AI)‐based endoscopy, and/or molecular markers (DNA methylation: BARHL2, MINT31, TET1, miR‐148a, miR‐124a‐3, NKX6‐1; mutations: TP53; and microsatellite instability) in diagnosing GIN lesions. We enrolled 24,388 patients who underwent endoscopy, and 71 patients were diagnosed with GIN lesions. Thirty‐two cases of endoscopic submucosal dissection (ESD) in 71 GIN lesions and 32 endoscopically resected tissues were assessed by endoscopists, AI, and molecular markers to identify benign or malignant lesions.

**Results:**

The board‐certified endoscopic physicians group showed the highest accuracy in the receiver operative characteristic curve (area under the curve [AUC]: 0.931), followed by a combination of AI and miR148a DNA methylation (AUC: 0.825), and finally trainee endoscopists (AUC: 0.588).

**Conclusion:**

AI with miR148s DNA methylation‐based diagnosis is a potential modality for diagnosing GIN.

## Graphical abstract

This study aimed to compare and evaluate the diagnostic sensitivity and specificity of physician‐performed endoscopy, AI‐based endoscopy, and/or molecular markers in detecting gastrointestinal neoplasms (GIN). We believe that our study makes a significant contribution to the literature because despite the availability of both endoscopic and histologic diagnosis, differentiating between a benign and malignant lesions is still challenging; some lesions are classified indeterminately as GIN. AI diagnosis is gaining popularity because it enables the non‐invasive diagnosis of the presence, site, and extent of lesions. Further, we believe that this paper will be of interest to the readership of your journal because we observed that the accuracy for GIN diagnosis from the combination of miR‐148a and AI was high.

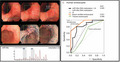

## 1 INTRODUCTION

Gastric cancer has the sixth highest incidence and the third most common cause of death from cancer worldwide.^1^ The World Health Organization estimates that gastric cancer accounted for 1,033,701 new cases and 782,685 deaths worldwide in 2018. Tremendous geographic variation exists regarding the incidence of gastric cancer worldwide. Disease incidence is low in North America and Northern Europe and highest in Asian countries (ie, Mongolia, Japan, and South Korea). The highest death rates were recorded in Western Asian countries (Iran, Turkmenistan, and Kyrgyzstan).

Endoscopic screening in Asian countries has reduced gastric cancer mortality.^2^ Early diagnosis increases the five‐year survival rate to >90%. However, early gastric cancer may be difficult to endoscopically diagnose (4.6–25.8% false‐negative rates).^3^, ^4^, ^5^, ^6^ Candidate molecular markers (ie, methylation, mutation, and microsatellite instability [MSI]) have been reported as accurate markers to detect early gastric cancer. Artificial intelligence (AI)‐based endoscopy has vast medical applications.

We aimed to compare and evaluate the diagnostic sensitivity and specificity of physician‐performed endoscopy, AI‐based endoscopy, and/or molecular markers in detecting gastric indefinite dysplasia (GIN).

## 2 MATERIALS AND METHODS

### 2.1 Patients, endoscopy, clinical samples, and DNA extraction

We enrolled a total of 24,388 patients who underwent endoscopy between April 2010 and March 2013 at St. Marianna University School of Medicine Hospital, Japan. A total of 6788 patients underwent gastric biopsy, and 71 underwent gastric biopsy after EGD for GIN (Category 2 according to the Vienna Classification of GIN). Under informed consent with patient and their family, 32 patients agreed to endoscopic resection for detail and accurate diagnosis but also treatment. We successfully performed endoscopic submucosal resection (ESD) in all cases by two expert (board‐certified) endoscopists, and 32 endoscopically resected tissues were used as a reference for the final pathological diagnosis. GIN diagnosis was classified according to (a) few atypical cells, (b) erosion and/or inflammation, and (c) tissue damage.^7^, ^8^ The EGD was performed by endoscopists, including both trainee and board‐certified physicians using a single gastrointestinal endoscope (GIF H260, GIF Q260, and GIF H‐260Z; Olympus Medical Systems, Co., Ltd.). The endoscopically resected tissues of the GIN lesions (histologic specimen) were fixed in formalin, and the histopathological characteristics of the studied samples were paraffin‐embedded. Genomic DNA of 32 samples was extracted from each FFPE tissue using the standard phenol/chloroform method. This study was reviewed and approved by the Ethics Committee of the St. Marianna University School of Medicine (No. #3345).

### 2.2 Candidate molecular marker analysis

Bisulfite polymerase chain reaction (PCR) and quantitative promoter DNA methylation analysis of pyrosequencing of candidate genes (BARHL2, MINT31, TET1, miR‐148a, miR‐124a‐3, NKX6‐1) were performed using an EpiTect Bisulfite kit (QIAGEN) and Pyromark Advanced Q24 system (Qiagen). Pyrosequencing quantitatively measures the methylation status of several CpG sites in each gene promoter. These adjacent sites usually show highly concordant methylation patterns. Therefore, the mean percentage of methylation at the detected sites was used as a representative value for the gene promoter. All primers and protocols were based on previous reports.^9^, ^10^, ^11^, ^12^, ^13^, ^14^ TP53 mutation analysis was performed and decided using immunohistochemistry (Anti‐TP53 [DO‐7] antibody). MSI analysis also was performed as previously described.^15^

### 2.3 Constructing a convolution neural network (CNN) algorithm

To construct an AI‐based diagnostic system, we used a deep neural network architecture called the single‐shot multi‐box detector (SSD) (http s://arxi v.org/abs/1512.0232 5), without altering its algorithm. SSD is a deep CNN that consists of ≥16 layers. The Caffe deep learning framework was then used to train, validate, and test the CNN. All CNN layers were fine‐tuned using stochastic gradient descent with a global learning rate of 0.0001. Each image was resized to 300 pixels ´ 300 pixels. The bounding box was also resized accordingly to optimize CNN analysis. These values were set up by trial and error to ensure that all data were compatible with SSD (AI Medical Service Inc.).

### 2.4 Outcome measures of AI‐based detection and diagnosis

A total of 2961 images from 32 cases were collected. There were some images that were unsuitable for consideration (GIN lesion was not in the image, out of focus images, blurry images, halation affect). Thus, two expert (board‐certified) endoscopist carefully selected 248 images finally that showed the GIN lesion without any issues.

ESD in all cases was performed by two expert (board‐certified) endoscopist. When the CNN detected a gastric mucosal abnormality in the lesion for all images, the CNN encodes a disease name (non‐tumorous lesion or tumorous lesion [cancer and/or adenoma]) and its position. The detected lesion was identified by a yellow rectangular frame on the endoscopic images, and the degree of reliability was calculated according to the measured result of the CNN.

### 2.5 Statistical analysis

All statistical analyses were performed using the SPSS for Windows (v.12. (SPSS, Inc.) and PRISM for Windows (v.7. (GraphPad Software). All reported *p*‐values were two‐sided, and statistical significance was set at *p* < 0.05. We computed the median DNA methylation value and range for each sample, and we defined the receiver operating characteristic (ROC) curve using SPSS. The z‐score analysis was used to normalize the methylation levels of several genes, microsatellite instability (MSI), TP53 gene mutation status, AI diagnosis, and endoscopist's diagnosis in each sample. The z‐score of the methylation for each gene was calculated as follows: z‐score = (methylation level of each sample – mean value of methylation level)/standard deviation of methylation level. In this analysis, a z‐score >0 indicates that the methylation level is greater than the mean value for the population.

## 3 RESULTS

### 3.1 Clinical characteristics and endoscopic images of all cases, lesions

**FIGURE 1:**
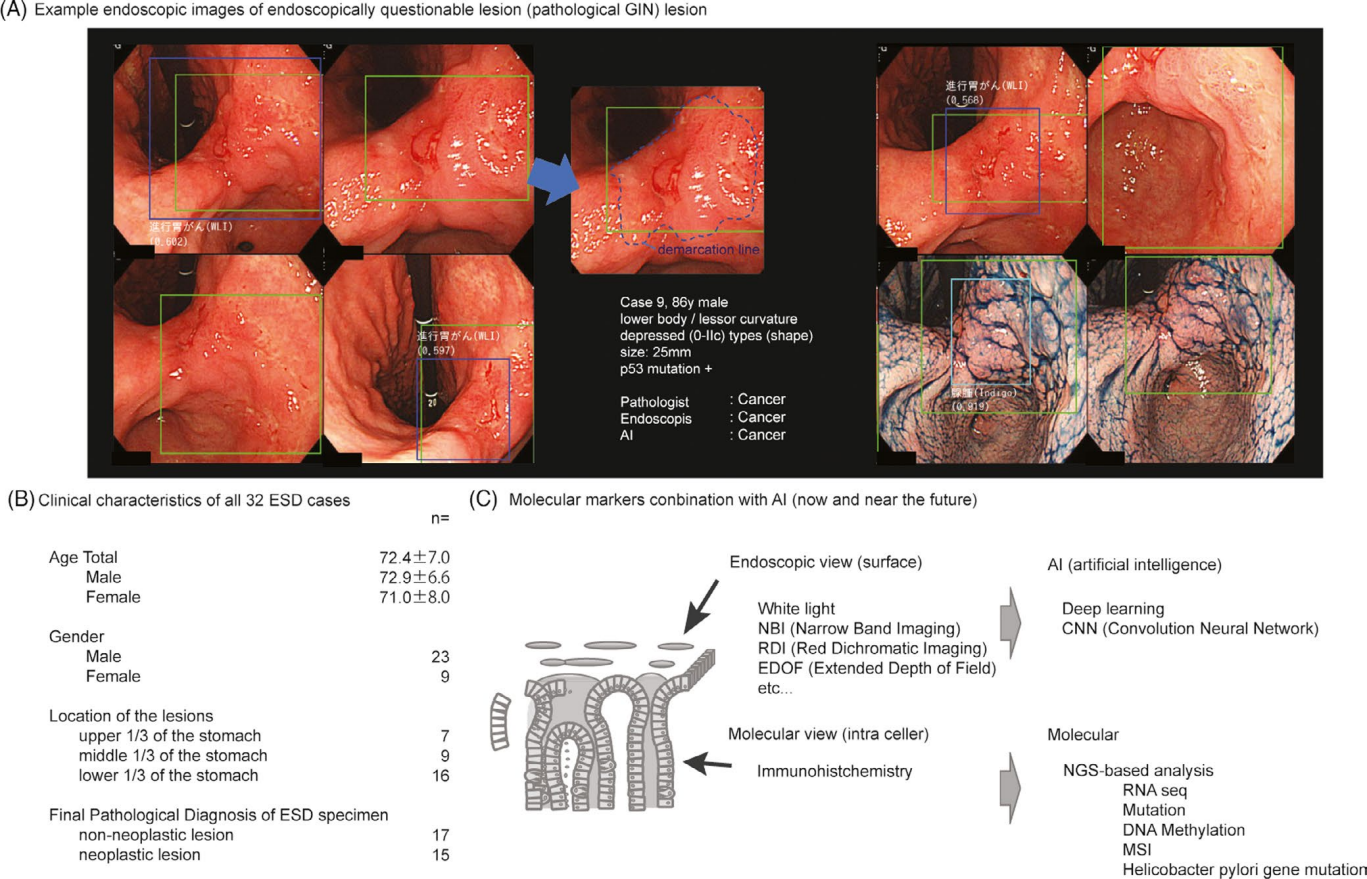
(A) Example endoscopic images of an endoscopically questionable lesion (pathological gastric indefinite for dysplasia lesion) (case 9). Artificial intelligence (AI) was used to detect the abnormal lesion, showing a blue square and diagnosed it based on yellow square area. An expert endoscopist decided the line on the abnormal lesion showing blue dots as a demarcation line. (B) Clinical characteristics of all 32 cases of endoscopic submucosal dissection (includes inflammation, high grade dysplasia, and cancer). (C) Schema for comparing the diagnostic tool between conventional endoscopy with immunohistochemistry, and AI‐based endoscopy with molecular markers

All endoscopic images of abnormal (indeterminate) lesions from the 32 cases of ESD histopathologically confirmed GIN were analyzed for AI diagnosis. Examples of the endoscopic images are shown in Figure 1A. The clinical characteristics of all cases are shown in Figure 1B and Table 1.

**TABLE 1:** Detail results of the clinical characteristics, molecular markers (immunohistochemistry, microsatellite instability, and methylation), and artificial intelligence diagnosis in 32 cases of endoscopic submucosal dissection

Gender	Age	Location		Tumor shape	With ulcer	Tumor size	Mutation	MSI	Microsatellite markers (Allele 1/ Allele 2)					DNA methylation markers (%)							AI diagnosis	Pathological findings after ESD endoscopic treatment
			Side	Macroscopic types	(UL)	(mm)	P53		BAT−25	BAT−26	NR−21	NR−24	MONO−27	BARHL2	P16	MINT31	TET1	miR148a	miR124a3	NKX6‐1		
Male	59	L	GC	Slightly elevated		12	**Mut**		(ー)	(ー)	(ー)	(ー)	(ー)	ー	8	ー	21	24	38	74	TURE	**Neoplastic lesion**
Female	69	L	AW	Depressed	−	20	**Mut**	MSH‐H	**(+)**	(ー)	(ー)	(ー)	**(+)**	81	7	1	11	14	51	91	FALSE	Non‐neoplastic lesion
Male	76	M	AW	Flat		22	WT		(ー)	(ー)	(ー)	(ー)	(ー)	ー	ー	9	29	16	29	2	TURE	Non‐neoplastic lesion
Male	69	M	AW	Depressed	−	15	**Mut**		(ー)	(ー)	(ー)	(ー)	(ー)	41	7	11	18	28	33	40	FALSE	Non‐neoplastic lesion
Male	72	L	PW	Depressed	+	25	**Mut**		(ー)	(ー)	(ー)	(ー)	(ー)	30	6	6	13	24	32	42	TURE	**Neoplastic lesion**
Male	70	L	GC	Depressed	−	10	WT	MSH‐L	(ー)	**(+)**	(ー)	(ー)	(ー)	ー	1	ー	18	20	17	76	FALSE	Non‐neoplastic lesion
Female	55	L	AW	Depressed	−	8	WT		(ー)	(ー)	(ー)	(ー)	(ー)	53	6	13	14	23	37	39	FALSE	Non‐neoplastic lesion
Female	73	M	LC	Depressed	+	18	WT		(ー)	(ー)	(ー)	(ー)	(ー)	44	4	7	15	23	40	57	FALSE	Non‐neoplastic lesion
Male	86	L	LC	Depressed	−	25	**Mut**		(ー)	(ー)	(ー)	(ー)	(ー)	64	3	1	4	28	29	44	TURE	**Neoplastic lesion**
Male	69	M	GC	Slightly elevated		19	WT		(ー)	(ー)	(ー)	(ー)	(ー)	34	4	15	11	15	38	52	FALSE	Non‐neoplastic lesion
Male	71	U	PW	Depressed	−	15	WT		(ー)	(ー)	(ー)	(ー)	(ー)	ー	ー	ー	1	10	38	ー	TURE	Non‐neoplastic lesion
Male	83	U	LC	Flat		12	WT		(ー)	(ー)	(ー)	(ー)	(ー)	49	1	1	13	15	66	96	FALSE	Non‐neoplastic lesion
Male	60	L	PW	Depressed	+	20	WT		(ー)	(ー)	(ー)	(ー)	(ー)	ー	ー	ー	3	17	66	96	TURE	**Neoplastic lesion**
Male	68	L	LC	Flat		10	**Mut**		(ー)	(ー)	(ー)	(ー)	(ー)	98	7	16	10	18	50	65	FALSE	Non‐neoplastic lesion
Male	70	L	PW	Depressed	−	8	**Mut**	MSH‐L	**(+)**	(ー)	(ー)	(ー)	(ー)	ー	ー	ー	73	28	56	ー	FALSE	Non‐neoplastic lesion
Male	75	L	AW	Slightly elevated		20	**Mut**		(ー)	(ー)	(ー)	(ー)	(ー)	ー	ー	ー	77	22	85	ー	TURE	**Neoplastic lesion**
Female	70	M	GC	Flat		12	WT		(ー)	(ー)	(ー)	(ー)	(ー)	ー	ー	ー	2	18	56	27	FALSE	Non‐neoplastic lesion
Female	71	L	AW	Depressed	−	20	WT		(ー)	(ー)	(ー)	(ー)	(ー)	39	ー	7	1	17	1	ー	FALSE	Non‐neoplastic lesion
Male	74	U	LC	Slightly elevated		10	WT		(ー)	(ー)	(ー)	(ー)	(ー)	ー	ー	0	ー	22	ー	ー	TURE	**Neoplastic lesion**
Female	78	U	LC	Slightly elevated		28	WT		(ー)	(ー)	(ー)	(ー)	(ー)	ー	ー	ー	1	24	83	ー	TURE	**Neoplastic lesion**
Male	71	M	LC	Depressed	−	12	**Mut**		(ー)	(ー)	(ー)	(ー)	(ー)	ー	ー	ー	1	22	ー	ー	Undecidable	Non‐neoplastic lesion
Female	78	U	AW	Depressed	−	10	WT		(ー)	(ー)	(ー)	(ー)	(ー)	98	6	0	13	14	71	58	FALSE	Non‐neoplastic lesion
Female	81	U	PW	Depressed	−	10	WT		(ー)	(ー)	(ー)	(ー)	(ー)	24	7	3	16	26	36	41	TURE	Non‐neoplastic lesion
Male	72	L	PW	Depressed	−	9	WT		(ー)	(ー)	(ー)	(ー)	(ー)	ー	ー	ー	3	20	18	ー	TURE	**Neoplastic lesion**
Male	81	L	LC	Slightly elevated		15	**Mut**		(ー)	(ー)	(ー)	(ー)	(ー)	52	16	0	12	24	48	44	TURE	**Neoplastic lesion**
Male	78	U	PW	Depressed	−	12	WT		(ー)	(ー)	(ー)	(ー)	(ー)	99	4	13	14	17	50	57	FALSE	Non‐neoplastic lesion
Male	79	L	PW	Depressed	−	10	WT		(ー)	(ー)	(ー)	(ー)	(ー)	ー	1	1	7	24	50	99	TURE	**Neoplastic lesion**
Male	73	M	PW	Depressed	−	10	**Mut**		(ー)	(ー)	(ー)	(ー)	(ー)	ー	3	0	8	16	49	ー	TURE	**Neoplastic lesion**
Male	74	L	AW	Depressed	+	14	WT		(ー)	(ー)	(ー)	(ー)	(ー)	ー	ー	ー	7	20	2	ー	TURE	**Neoplastic lesion**
Male	66	L	LC	Slightly elevated		12	WT		(ー)	(ー)	(ー)	(ー)	(ー)	ー	ー	ー	10	30	12	ー	TURE	**Neoplastic lesion**
Female	64	L	AW	depressed	−	10	WT		(ー)	(ー)	(ー)	(ー)	(ー)	99	ー	ー	1	22	84	ー	TURE	**Neoplastic lesion**
Male	81	L	LC	Depressed	−	9	**Mut**		(ー)	(ー)	(ー)	(ー)	(ー)	50	12	0	14	28	34	39	TURE	**Neoplastic lesion**

Note

Gray shaded area are title of diagnostic factors.

All bold values are important factors for diagnosis.

TRUE, AI diagnosed as a neoplasitc lesion, FALSE, AI diagnosed as a non‐neoplastic lesion.

AW, anterior wall; BARHL2, BarH like homeobox 2; GC, greater curvature; L, lower 1/3 of the stomach; LC, lesser curvature; M, middle 1/3 of the stomach; MINT31, methylation in tumor 31; miR148a, microRNA 148a; MSI‐H, microsatellite instability high; MSI‐L, microsatellite instability low; Mut, mutation; NKX6‐1, NKX homeobox 1; p16, cyclin‐dependent kinase inhibitor 2A; PW, posterior wall; TET1, Tet methylcytosine dioxygenase 1; U, upper 1/3 of the stomach; WT, wild type.

### 3.2 Clinical diagnostic ability of AI, molecular markers, and endoscopist

**FIGURE 2:**
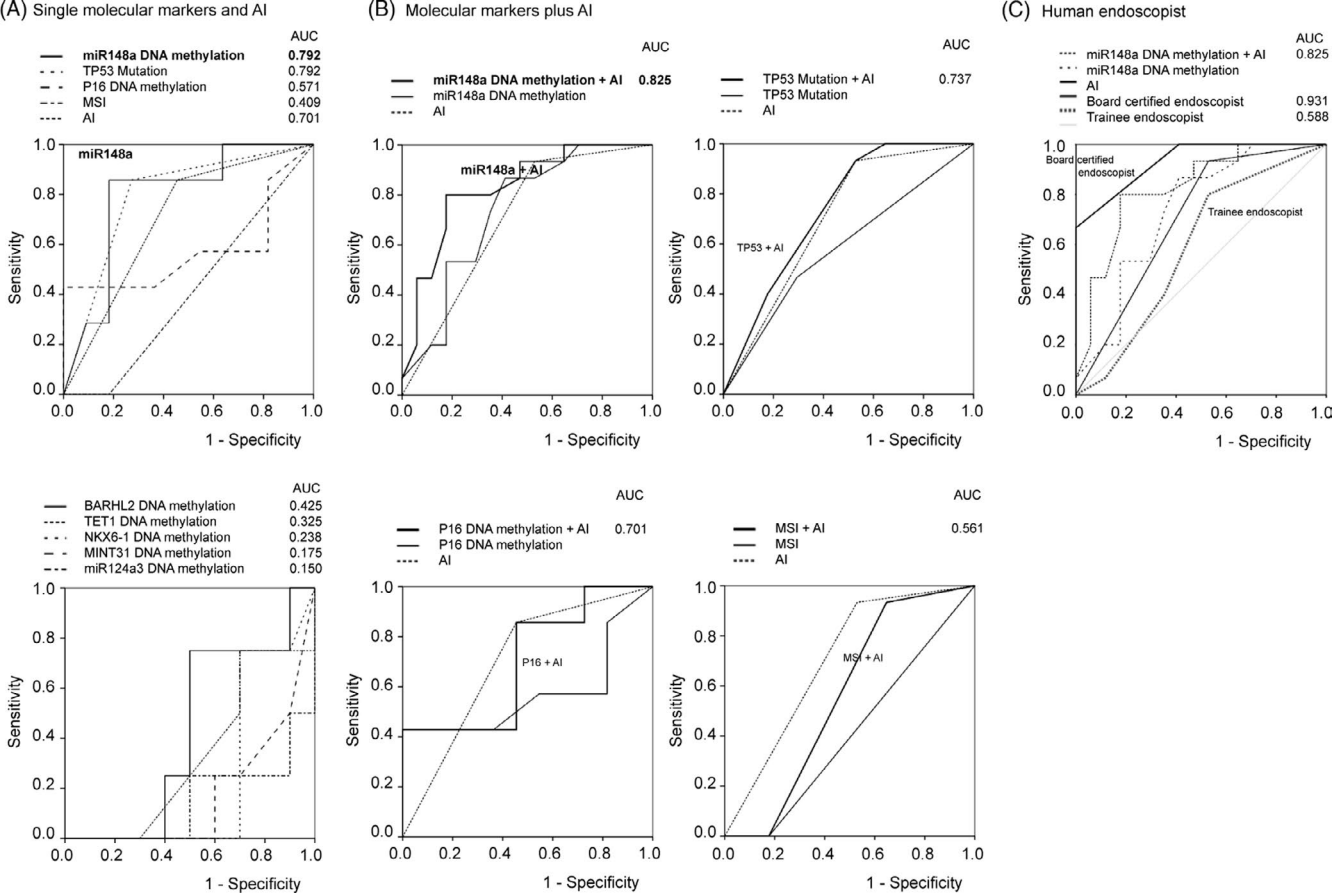
Receiver operating curve (ROC) of molecular markers (DNA methylation: BARHL2, MINT31, TET1, miR‐148a, miR‐124a‐3, NKX6‐1; mutation: TP53; and microsatellite instability), artificial intelligence (AI), and endoscopist. All diagnostic abilities were calculated using ROC and area under the curve based on the z‐score of each factor. (A) Single molecular markers and AI in 32 gastric indefinite for dysplasia (GIN) lesions. (B) Combination of molecular markers with AI in 32 GIN lesions. (C) Comparison of the miR148a methylation/AI combination and endoscopist (board certificated endoscopists [*n* =3] and trainee endoscopists [*n* =3])

We first calculated the individual diagnostic ability of AI, molecular markers, and two endoscopists using the area under the curve (AUC) of the ROC curve in Figure 2.

**FIGURE 3:**
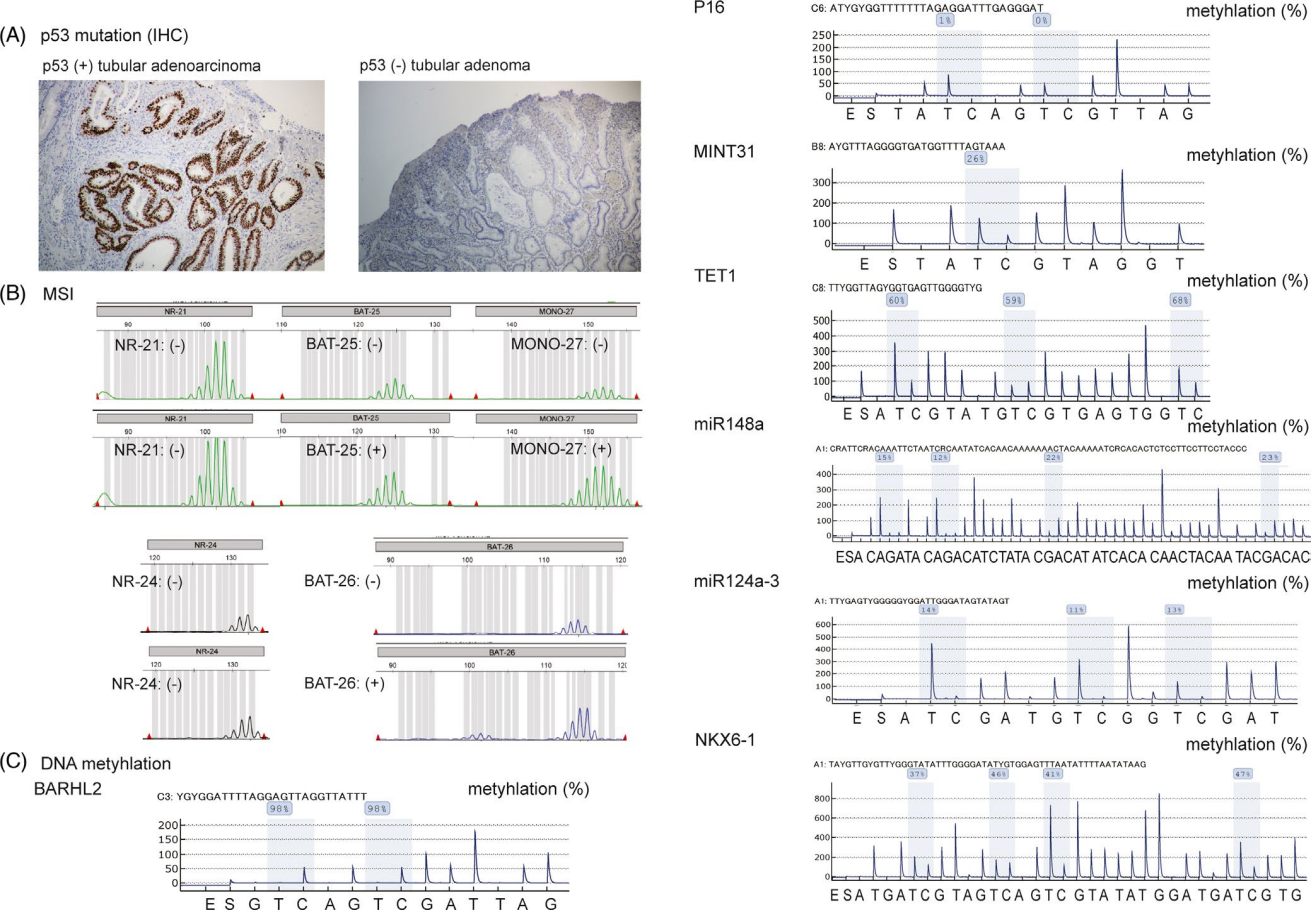
(A) Example of the TP53 positive/negative immunohistochemistry (IHC) staining. (B) Microsatellite instability (MSI) analysis. Example MSI‐H/MSI‐L analysis using 5 MSI markers (BAT‐25, BAT‐26, NR‐21, NR‐24, and MONO‐27). (C) DNA methylation analysis using quantitative pyrosequencing using 7 candidate molecular markers (BARHL2, P16, MINT31, TET1, miR148a, miR124a3, and NKX6‐1)

AI detected 96.9% of the cases (31/32) of GIN lesions and diagnosed as TURE (neoplastic lesion) or FALSE (non‐neoplastic lesion). We observed a sensitivity of 75.0% and specificity of 50% (AUC, 0.701) for the AI diagnostic power referenced from pathological findings after ESD (Figure 2A). In genetic alteration of TP53 gene mutation analysis, we found 12 positive cases via immunohistochemistry (IHC) staining using a TP53 antibody (37.5%, 12/32) even in non‐neoplastic lesions (Figure 3A, Table 1). In the MSI analysis, we found MSI‐H/MSI‐L cases (9.4%, 3/32) in only non‐neoplastic lesions using 5 MSI markers (BAT‐25, BAT‐26, NR‐21, NR‐24, MONO‐27) (Figure 3B, Table 1). On DNA methylation analysis, we found a sensitivity of 75.0% and specificity of 50.0% for BARHL2 (AUC, 0.425); 75.0% and 40.0%, respectively, for P16 (AUC, 0.701); 25.0% and 40.0%, respectively, for MINT31 (AUC, 0.175); 75.0% and 40.0%, respectively, for TET1 (AUC, 0.325); 100.0% and 80.0%, respectively, for miR148a (AUC, 0.825); 50.0% and 10.0%, respectively, for miR124a3 (AUC, 0.150); and 50.0% and 30.0%, respectively, for NKX6‐1 (AUC, 0.238) (Figure 3C, Table 1).

The best AUC was for the biomarker miR148a DNA methylation (AUC, 0.792), followed by AI (AUC, 0.701) (Figure 2A). We then calculated the diagnostic ability of AI when combined with the other two factors. The best AUC was for AI + miR148a (AUC: 0.825) as shown in Figure 2B. Interestingly, the diagnostic power of the AI + miR148a combination was located between the diagnostic power of board‐certified endoscopists (AUC: 0.931) and trainees (AUC: 0.588) (Figure 2C).

## 4 DISCUSSION

Gastric cancer has the sixth highest incidence and the third most common cause of death from cancer worldwide. However, early gastric cancer may be difficult to endoscopically diagnose. Candidate molecular markers (ie, methylation, mutation, and MSI) have been reported as accurate markers to detect early gastric cancer.

Image recognition using AI has dramatically improved due to innovative technologies such as machine learning and deep learning. These techniques are now being applied to gastrointestinal endoscopy worldwide. AI has high diagnostic accuracy for esophageal, gastric, and colorectal cancers.^16^, ^17^, ^18^ However, AI has been mostly used to identify irregular or malignant lesions. Qualitative investigations for a comprehensive diagnosis to facilitate appropriate therapy remain limited. Despite direct visualization of the lesion during endoscopy, findings can be difficult to classify as benign irregular lesions (ulcers, infections, or other factors) or malignant even in using additional narrow band imaging, red dichromatic imaging, extended depth of field, and magnified view functions (Figure 1C). Post‐endoscopy, biopsy remains essential for clinicians for a definitive diagnosis and to formulate a treatment plan.^19^ Despite the availability of both endoscopic and histologic diagnosis, differentiating between benign and malignant lesions is still challenging; some lesions are classified indeterminately as GIN.^20^ One reason is the difficulty of evaluating the entire lesion pathologically using only a fragment of tissue. In some cases, patient consent is obtained to perform minimally invasive ESD for both therapeutic and diagnostic purposes.^21^, ^22^, ^23^ This makes it possible to perform a histological assessment of the entire lesion to determine whether it is benign or malignant. Conventionally, it is ideal to assess ESD.

Mechanisms of malignant transformation due to genetic abnormalities include driver gene mutations and the accumulation of passenger gene abnormalities due to epigenetic alterations including DNA and microRNA methylation.^24^, ^25^, ^26^ In gastric cancer, the accumulation of abnormalities due to epigenetic gene alterations from *Helicobacter pylori* infection is important in tumorigenesis.^27^, ^28^, ^29^, ^30^, ^31^, ^32^ Multiple studies have investigated the clinical applications of these candidate molecular markers for diagnosis; while the “risk and predictive diagnoses” for malignant gastric transformation has been achieved, its practical applications to diagnose the presence, site, and extent of lesions have not been sufficiently realized.

AI diagnosis, which involves assessments based on various data on the surface of the lesion, is gaining popularity because it enables the non‐invasive diagnosis of the presence, site, and extent of lesions (Figure 1C). The diagnostic capacity of AI for gastric cancer has been reported superior to that of endoscopists in training, and at par with specialists (board‐certified endoscopic physicians). We used AI diagnosis alone or in combination with molecular markers (methylation, mutation, MSI) and endoscopic diagnosis in 32 patients who underwent ESD with a preoperative pathological diagnosis of GIN to perform retrospective single factor and multifactor assessments with ROC. We observed that the accuracy for GIN diagnosis from the combination of miR‐148a and AI was extremely high. The AUC results were second only to certified endoscopists for diagnosing GIN lesions. This high accuracy may be due to the combined benefits of AI (surface information) in its ability to diagnose the presence, site, and range of the entire lesion and the accuracy of the molecular marker (cell information). This overcomes the limitation of histologic diagnosis that uses only limited tissue samples (Figure 1C).

Digital technology is already expanding in the medical field, even in the clinical diagnosis of gastric cancer. Clinicians often use endoscopy for diagnosis not only with white light, but also with a digital magnified function and narrow banding imaging function. Indigo carmine staining is also a helpful tool for diagnosis; however, it does not have enough diagnostic power for gastric cancer, especially for tiny lesions and any artifact lesions (biopsy scar, *H*. *pylori* infection, ulceration, inflammation, or drug effect) even when used by an expert endoscopist. AI diagnosis may have the potential to support endoscopists of all skill levels and may also be helpful to shorten the learning curve for trainee endoscopists. Moreover, we speculate that a combination of molecular markers may not only be useful for a more detailed assist diagnosis, but may also be an auxiliary tool for therapeutic strategies.

Our study has several limitations. This study only examined a relatively small sample size using a limited number of genetic markers. Going forward, more studies involving a comprehensive gene search are needed.

## CONFLICT OF INTEREST

The authors disclose no conflict of interest.

## AUTHOR CONTRIBUTIONS

Watanabe Y, Oikawa R, and Matsuo Y designed and coordinated the study and performed the experiments, acquired, and analyzed data; Watanabe Y, Oikawa R, Agawa S, Matsuo Y, Tada T, and Yamamoto H interpreted the data; Watanabe Y, Oikawa R and Futagami S validated mutant strains using bioinformatics approach; Futagami S, Oda I, Yamamoto H, and Itoh F wrote or edited the manuscript; all authors approved the final version of the article.

## APPROVAL CODE ISSUED BY THE INSTITUTIONAL REVIEW BOARD (IRB)

This study was reviewed and approved by the Ethics Committee of the St. Marianna University School of Medicine (No. #3345).

## Data Availability

The data that support the findings of this study are available from the corresponding author upon reasonable request.
